# Communication Between Cardiomyocytes and Fibroblasts During Cardiac Ischemia/Reperfusion and Remodeling: Roles of TGF-β, CTGF, the Renin Angiotensin Axis, and Non-coding RNA Molecules

**DOI:** 10.3389/fphys.2021.716721

**Published:** 2021-09-03

**Authors:** Raúl Flores-Vergara, Ivonne Olmedo, Pablo Aránguiz, Jaime Andrés Riquelme, Raúl Vivar, Zully Pedrozo

**Affiliations:** ^1^Advanced Center for Chronic Diseases (ACCDiS), Facultad de Ciencias Químicas y Farmacéuticas & Facultad de Medicina, Universidad de Chile, Santiago de Chile, Chile; ^2^Programa de Fisiología y Biofísica, Instituto de Ciencias Biomédicas (ICBM), Facultad de Medicina, Universidad de Chile, Santiago de Chile, Chile; ^3^Programa de Fisiopatología, Instituto de Ciencias Biomédicas (ICBM), Facultad de Medicina, Universidad de Chile, Santiago de Chile, Chile; ^4^Red para el Estudio de Enfermedades Cardiopulmonares de alta letalidad (REECPAL), Universidad de Chile, Santiago de Chile, Chile; ^5^Escuela de Química y Farmacia, Facultad de Medicina, Universidad Andrés Bello, Viña del Mar, Chile; ^6^Departamento de Química Farmacológica y Toxicológica, Facultad de Ciencias Químicas y Farmacéuticas, Universidad de Chile, Santiago de Chile, Chile; ^7^Programa de Farmacología Molecular y Clínica, Instituto de Ciencias Biomédicas, Facultad de Medicina, Universidad de Chile, Santiago de Chile, Chile

**Keywords:** myocardial infarction, ischemia/reperfusion, crosstalk, fibroblasts, cardiomyocytes

## Abstract

Communication between cells is a foundational concept for understanding the physiology and pathology of biological systems. Paracrine/autocrine signaling, direct cell-to-cell interplay, and extracellular matrix interactions are three types of cell communication that regulate responses to different stimuli. In the heart, cardiomyocytes, fibroblasts, and endothelial cells interact to form the cardiac tissue. Under pathological conditions, such as myocardial infarction, humoral factors released by these cells may induce tissue damage or protection, depending on the type and concentration of molecules secreted. Cardiac remodeling is also mediated by the factors secreted by cardiomyocytes and fibroblasts that are involved in the extensive reciprocal interactions between these cells. Identifying the molecules and cellular signal pathways implicated in these processes will be crucial for creating effective tissue-preserving treatments during or after reperfusion. Numerous therapies to protect cardiac tissue from reperfusion-induced injury have been explored, and ample pre-clinical research has attempted to identify drugs or techniques to mitigate cardiac damage. However, despite great success in animal models, it has not been possible to completely translate these cardioprotective effects to human applications. This review provides a current summary of the principal molecules, pathways, and mechanisms underlying cardiomyocyte and cardiac fibroblast crosstalk during ischemia/reperfusion injury. We also discuss pre-clinical molecules proposed as treatments for myocardial infarction and provide a clinical perspective on these potential therapeutic agents.

## Introduction

Cardiovascular diseases are the leading cause of death worldwide. Acute myocardial infarction (AMI) is responsible for a significant share of this mortality, resulting in more than 2.4 million deaths each year in the United States (Townsend et al., 2016). AMI usually occurs due to sudden obstruction of a main branch of the coronary artery (Sen et al., 2017). This blockage brings about an abrupt drop in cardiac blood flow and, consequently, a decrease in nutrient and oxygen supply, leading to non-apoptotic cell death and release of pro-inflammatory mediators by dead and damaged cardiomyocytes (CM; Reed et al., 2017). Re-establishing blood supply to the ischemic zone (cardiac reperfusion) is crucial to minimize tissue injury. Paradoxically, however, restored flow can cause further damage, a phenomenon known as reperfusion injury. The sudden increase in oxygen levels induces production and release of reactive oxygen species (ROS; Granger and Kvietys, 2015), which, in addition to initiating a pro-inflammatory process, accounts for a large proportion of the cardiac damage. Myocardial infarction (MI) is ultimately the result of the injury induced by ischemia/reperfusion (IR) to the CM and surrounding heart cells (Sacks et al., 2018).

Post-AMI repair involves a complex series of finely-coordinated events. The inflammatory phase begins with sterile acute inflammation accompanied by immune cell infiltration into the heart. The inflammatory cells digest and eliminate dead cells and damaged extracellular matrix (ECM) tissue. Afterward, cardiac fibroblasts (CF) guide the reparative and proliferative phase. These cells can proliferate, release soluble factors into the environment, and differentiate into specialized cells called as myofibroblasts (MF), which in turn synthesize large amounts of ECM proteins to maintain the structural integrity of the tissue. This phase is characterized by the resolution of inflammation and development of scar (Prabhu and Nikolaos, 2016).

When inflammation is not appropriately resolved, chronic remodeling takes place. This remodeling leads to large areas of scarring and collagen deposits in the infarcted tissue, causing rigidity of the ventricular wall, loss of contractile force, and reduced systolic volume. These changes may eventually lead to heart failure (Berezin and Berezin, 2020). Protecting the heart from this pathological reparative process is vital, and the pursuit of new therapeutic targets to avoid IR injuries and their sequelae remains a crucial challenge for the scientific community.

Several studies have explored therapeutic approaches for cardioprotection. The most-studied strategies include ischemic postconditioning, drug administration, and physical interventions (such as electrical nerve stimulation; Davidson et al., 2019). However, despite success in animal models, it has not been possible to replicate positive results in humans, possibly because of the multiple comorbidities and comedications common in patients (Heusch and Gersh, 2017).

There has been great interest in CM-CF crosstalk as a therapeutic target, as CF exert a protective effect on CM during IR injury (Abrial et al., 2014). Evidence shows that both CF and MF mediate CM structure and function to regulate cardiac tissue structure, electrical characteristics, and mechanical activity under physiological and pathological conditions such as IR injury (Lyu et al., 2015; Hooshdaran et al., 2017). In any case, our knowledge of CM-CF crosstalk CF in IR injury is incomplete.

Thus, in this review, we will examine the main soluble factors released by CM and CF and their probable roles during IR, as well as relevant signaling pathways and biological effects. We will also discuss pre-clinical drugs to modulate cardiac cell response during AMI. More extensive knowledge of CM-CF crosstalk will bring us closer to comprehensively understanding the pathological mechanisms that affect cardiac function during IR injury.

## Communication Between Cardiomyocytes and Cardiac Fibroblasts During Ischemia/Reperfusion Injury

Crosstalk between cells is a crucial mechanism for maintaining the physiological metabolism, survival, and function of any tissue or organ. CM and CF, along with immune system and endothelial cells, release several kinds of molecules that regulate heart activity. CM-CF crosstalk, in parallel with pressure and volume overload, is of utmost importance for inducing myocardial remodeling after AMI. Three types of CM-CF cell communication have been described: paracrine signaling, direct cell-to-cell interactions, and ECM-mediated signaling (Pellman et al., 2016; Humeres and Frangogiannis, 2019). CM and CF produce and release autocrine/paracrine factors that allow interaction between the cells. The cells secrete growth factors for paracrine signaling, including transforming growth factor-β (TGF-β), fibroblast growth factor (FGF), connective tissue growth factor (CNN2/CTGF), angiotensin II (Ang II), insulin-like growth factor-1 (IGF-1), along with cytokines such as interlukin-1 (IL-1) and tumor necrosis factor alpha (TNF-α; Bang et al., 2015; Cartledge et al., 2015; Svystonyuk et al., 2015). Cadherins and connexins mediate direct cell-to-cell communication. Finally, mechanosensors such as integrins regulate indirect CM-CF interactions through ECM signaling (Okada et al., 2013; Perbellini et al., 2018; Ward and Iskratsch, 2020). Heart injuries, such as the insult triggered in AMI, may disturb the normal secretion of these factors and activate cell communication pathways that elicit CF-to-MF differentiation, CM hypertrophy, and cardiac remodeling.

## Molecules Released by Cardiomyocytes and Fibroblasts During IR

CM and CF release several types of soluble factors during IR injury to orchestrate the reparative process and support continuous function of the heart. The following molecules have been studied and reported as promising targets for understanding CM-CF crosstalk during IR, offering potential therapeutic strategies.

### Transforming Growth Factor-β

Several cell types have been recognized as sources of TGF-β in the heart including CM, endothelial cells, CF, and macrophages. TGF-β1 is necessary for the maturation of CM and physiological activity of CF (Huntgeburth et al., 2011; Vilahur et al., 2011; Johnston and Gillis, 2018). However, TGF-β1 also induces changes in cardiac cells that promote pathological remodeling, including cardiac fibrosis after AMI and CM hypertrophy, leading to contractile and electrical disorders, myocardial dysfunction, and sometimes sudden death (Dobaczewski et al., 2010; Wenzel et al., 2010).

TGF-β1 recognizes type I (TβRI) and type II (TβRII) membrane receptors. TGF-β1 binds to TβRI and induces heterodimerization with TβRII, which activates canonical TGF-β1 signaling through intracellular proteins called as SMAD. These phosphorylation-activated SMAD transcription factors interact with the common SMAD4 protein to form a transcriptional complex that binds to SMAD-specific recognition sites (SRE) on the promoter of TGF-β1-regulated genes in DNA. Furthermore, proteins, such as TAK1, RhoA, p38, NFkB, ERK, and PI3K/AKT, can be activated *via* TβRII through non-canonical TGF-β1 signaling pathways (Dobaczewski et al., 2011; Neuzillet et al., 2014).

It has been broadly demonstrated that TGF-β increases tissue damage, impairs cardiac function, and promotes ventricular remodeling after IR injury (Rainer et al., 2014; Liang et al., 2018; Liu et al., 2019b). Nonetheless, the mechanism that activates TGF-β during IR is unclear. ROS production may induce increased TGF-β1 release and activation in IR injury (Frangogiannis, 2017). Reduced TGF-β1 levels were reported in patients treated with N-acetylcysteine, indicating that TGF-β1 levels may be regulated by ROS availability (Talasaz et al., 2013). However, the reciprocal regulation of TGF-β1 and ROS has also been described (Liu and Desai, 2015).

It is interesting that both CM and CF synthesize and secrete TGF-β and express TβR, suggesting a potential for crosstalk between the cell types. Indeed, CM-CF crosstalk through TGF-β has been demonstrated during CM hypertrophy provoked by pressure overload (Cartledge et al., 2015) or mechanical stretch (Van Wamel et al., 2001). This hypertrophic effect depends on the release of this soluble mediator by CF. On the other hand, it is the TGF-β secreted by CM that is crucial for inducing collagen I secretion by CF in the presence of Ang II (Cartledge et al., 2015). Under baseline conditions, CF-conditioned medium induces CM hypertrophy (Moussad and Brigstock, 2000), and TGF-β1 released by MF regulates the expression and function of CM sodium and potassium ion channels, possibly pointing to a new mechanism that may at least partly explain the electrical remodeling during myocardial injury (Kaur et al., 2013).

Although there are few reports of TGF-β1-mediated crosstalk between CM and CF during IR injury, *in vitro* studies suggest that TGF-β1 released from CM during late hypoxia could inhibit CF migration (Shi et al., 2017). On the other hand, conditioned medium from endothelial cells transformed to CF-like cells during hypoxia/reoxygenation (H/R) provoked CM apoptosis and SMAD2 activation. These effects were prevented by TβRI inhibition (Sniegon et al., 2017), demonstrating the specificity of TGF-β in this cellular response. It has also been reported that TGF-β promotes apoptotic cell death *via* SMAD activation in adult rat CM (Schneiders et al., 2005) and that this pathway may contribute to CM death after AMI *in vivo* (Inman et al., 2002).

While the TGF-β1/SMAD3 signaling pathway induced fibrosis and apoptosis in cardiac cells subjected to H/R, miR-25 overexpression (Liu et al., 2016), glutamine treatment (Zhang et al., 2016), and mir-195-5p overexpression (Xie et al., 2020) protected cardiac cells from these deleterious effects by inhibiting the TGF-β1/SMAD3 pathway.

On the other hand, increased CM apoptosis and infarct size are also associated with p38 kinase activation *via* TAK1, possibly reflecting the involvement of non-canonical TGF-β/TAK1 signaling in CM death during IR injury (Marber et al., 2011; Wang et al., 2013). Moreover, cardiac hypertrophy after AMI is linked to TGF-β signaling pathway activation. In *in vivo*, specific blockade of TβRI by GW788388 attenuated collagen accumulation and reduced CM hypertrophy after MI (Tan et al., 2010). The TGFβ1-TAK1-p38 MAPK signaling pathway was found to be active after AMI, accompanied by an upregulation of cardiac hypertrophy markers, suggesting that this non-canonical pathway may be involved in cardiac hypertrophy after MI (Zhang et al., 2000; Matsumoto-Ida et al., 2006). However, whether TGF-β is released from CM or CF – or both, *via* redundant crosstalk pathway activation – is unclear.

Other studies in CM showed that TGF-β1 prevents cell death from anoxia/reoxygenation (Wang et al., 2016b) or H/R (Dandapat et al., 2008). Accordingly, TGF-β-induced protection may be related to ERK activation through the reperfusion injury salvage kinase (RISK) pathway (Hausenloy and Yellon, 2004).

The release of TGF-β during hypoxia and its influence on CF has also been explored. It has been reported that this process may be associated with reduced CF migration and CF-to-MF differentiation *via* transcription factor forkhead box O1 (FoxO1) expression (Vivar et al., 2016; Shi et al., 2017).

TGF-β is also involved in protecting CF from apoptosis induced by simulated IR (sIR) through canonical (SMAD3) and non-canonical (ERK1/2 and AKT) signaling pathways (Vivar et al., 2013). In addition, SMAD2 and SMAD3 CF knockout (KO) mice showed decreased numbers of MF and greater cardiac damage during AMI. TGF-β1/SMAD signaling, therefore, may play a key role in CF viability and cardiac tissue repair (Huang et al., 2019).

In addition to the classical effect of SMAD activation on gene expression, a relatively new aspect of microRNA (miRNA) regulation has been described. TGF-β1-activated SMAD bind to various miRNA promoters, enhancing or reducing their transcription. SMAD contribute to post-translational miRNA processing by association with the Drosha-complex, which is responsible for cutting pre-miRNA into its active forms (Blahna and Hata, 2012). After MI, TGF-β downregulates miR-29a expression and upregulates miR-21, and the synthesis of collagen and other ECM proteins involved in myocardial fibrosis is enhanced (Van Rooij et al., 2008; Liang et al., 2012).

**Figure 1:**
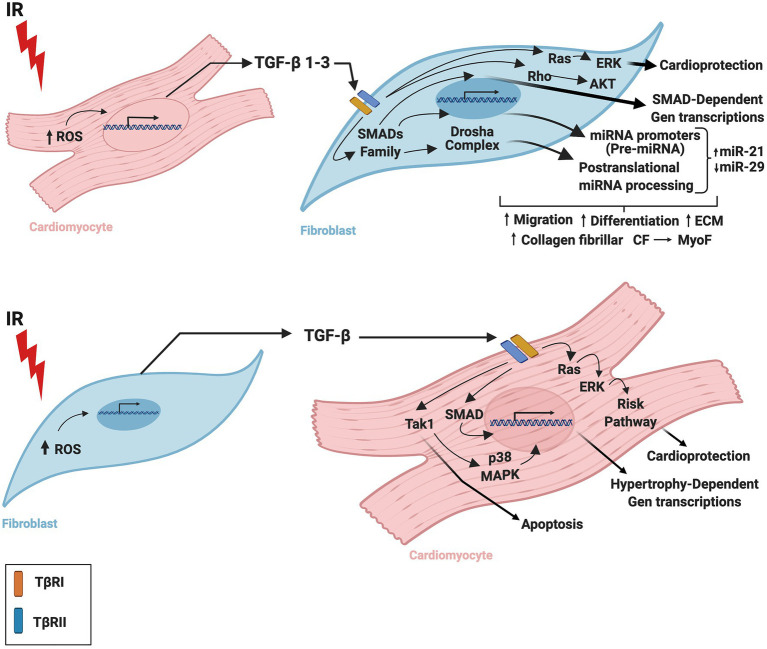
Role of TGF-β in the crosstalk between cardiomyocytes and fibroblasts during ischemia/reperfusion. TGF-β is secreted by cardiac fibroblasts and cardiomyocytes during ischemia-reperfusion inducing cardiac repair and remodeling. TGF-β is required for cardiac fibroblast differentiation and cardiomyocyte hypertrophy. IR, ischemia/reperfusion; TGF-β, transforming growth factor beta; ROS, reactive oxygen species; TβRI, transforming growth factor beta receptor I; TβRII, transforming growth factor beta receptor II; CM, cardiomyocytes; CF, cardiac fibroblasts; MyoF, myofibroblast. The figure was created using BioRender.com.

Although it is apparent that TGF-β has a crucial function in cardiac tissue, the TGF-β-related mechanisms by which CM and CF regulate one other during IR are still being elucidated. Insights into the complexity of these mechanisms and the contributions of cardiac cells to infarction-related upregulation of TGF-β may advance the design and development of drugs to regulate tissue remodeling or mitigate injury after IR. Figure 1 summarizes the CM-FB crosstalk regulated by TGF-β during cardiac IR.

### Connective Tissue Growth Factor

Connective tissue growth factor (CTGF), also referred to as CCN2, belongs to the CCN (acronym for Cyr61, CTGF, and Nov) family of matricellular proteins that regulate diverse aspects of cellular function such as inflammation, tissue repair, and fibrosis (Leask, 2010). It is well-known that CTGF is upregulated in a variety of fibrotic diseases, including cardiac pathologies (Jun and Lau, 2011). Indeed, cardiac CTGF expression is elevated in models of MI-induced heart failure (HF) and cardiac remodeling (Ahmed et al., 2004; Dean et al., 2005; Gabrielsen et al., 2007; Zhang et al., 2012), and CTGF protein levels have been correlated with the degree of myocardial fibrosis in HF patients (Koitabashi et al., 2007). Despite this evidence, the role of this molecule in cardiac fibrosis is still considered as controversial.

CTGF is a recognized ligand for a variety of receptors, such as epidermal growth factor receptor (EGFR; Alam et al., 2017), 6-phosphate/insulin-like growth factor 2 receptor (M6P/IGF-2-R), and lipoprotein receptor-related protein/α2-macroglobulin receptor (LRP; Blalock et al., 2012).

In the heart, CTGF mediates CF proliferation, migration, and adhesion (Kemp et al., 2004), CF-to-MF differentiation during cardiac fibrosis (Frazier et al., 1996), and CM hypertrophy (Hayata et al., 2008; Panek et al., 2009). Both CM and CF express and release CTGF in response to various stimuli including mechanical stretch, ROS, Ang II, TGF-β, and IR (Blom et al., 2002; Ahmed et al., 2004; Matsui and Sadoshima, 2004; Shakil Ahmed et al., 2011; Dorn et al., 2018; Zhao et al., 2019; Zhuang et al., 2019; Long et al., 2020; Aránguiz et al., 2021). Various signaling pathways are involved in CTGF upregulation, including SMAD, protein kinase C (PKC), ROS, RhoA, c-Jun NH2-terminal kinase (JNK), phosphatidylinositol 3-kinase (PI3K), and ERK (Matsui and Sadoshima, 2004). As several molecular signals regulate CTGF levels, most of them associated with compensatory responses after AMI, CTGF may act in an autocrine/paracrine manner in CM and CF during MI. Nevertheless, crosstalk between these cells remains to be completely understood, given the complexity of the signals. Furthermore, no CTGF-specific surface receptor has been identified to date.

After AMI, non-myocyte cells such as CF and endothelial cells show intense CTGF expression, mainly in granulation and scar tissue located in the interzone between necrotic and viable myocardium, as well as in myocardium distal to the ischemic zone (Ahmed et al., 2004; Dean et al., 2005). CTGF mRNA and proteins are also localized to the CM in the viable myocardium early after AMI (Dean et al., 2005). However, the function of CTGF during IR injury is still being elucidated.

It has been reported that hearts from transgenic mice with the cardiac-restricted overexpression of CTGF (Tg-CTGF), as well as hearts from wild-type mice perfused with recombinant hCTGF, demonstrate increased Akt/p70S6 kinase/GSK-3β pathway activity and induction of genes with known cardioprotective effects (Shakil Ahmed et al., 2011). Similarly, isolated adult CM treated with CTGF and subjected to H/R showed resistance to oxidative stress and cell death, also associated with Akt/p70S6 kinase/GSK-3β pathway activation (Moe et al., 2013), suggesting a cardioprotective role for CTGF during IR injury.

Despite limited information on CM-FC crosstalk, it has been observed that CTGF expression and release into the culture medium is elevated in CM stimulated with profibrotic factors such as TGF-β, endothelin-1, or Ang II. Furthermore, conditioned medium from these CM enhanced COL1A1 mRNA expression in CF, which was prevented with CTGF-neutralizing antibody (Koitabashi et al., 2007). In addition, sustained β-adrenergic receptor activation in CM significantly promoted CTGF synthesis and secretion, which acted in a paracrine manner to induce cell proliferation and synthesis of collagen I, collagen III, and α-SMA in CF (Nuamnaichati et al., 2018). Moreover, in a cardiac hypertrophy model, CTGF deletion from activated fibroblasts inhibited fibrotic remodeling, while deletion from CM had no effect (Dorn et al., 2018). *In vitro* experiments revealed that although efficiently secreted by both CF and CM, only fibroblast-derived CTGF is effective in fully activating CF. Therefore, it is possible that CF-secreted CTGF acts in an autocrine manner to modulate fibrosis in the heart (Dorn et al., 2018). On the other hand, CTGF overexpression in CM did not provoke cardiac fibrosis in an animal model but was rather associated with hypertrophic changes at a cellular level, which in turn was related to AKT and JNK pathway activation (Panek et al., 2009).

Interestingly, it has been recently reported that neonatal CM culture subjected to sIR showed upregulated CTGF expression that was dependent on the polycystin-1/AKT pathway. Furthermore, conditioned medium obtained from CM subjected to sIR enhanced CF activation in a paracrine manner, increasing migration, differentiation to MF, and COL1A1, TGF-β1, and CTGF expression (Aránguiz et al., 2021), suggesting that CTGF may contribute to CM-CF crosstalk during IR.

In sum, CTGF is secreted as an inactive pre-protein that requires proteolytic activation before initiating signaling (Kaasbøll et al., 2018). This molecule regulates multiple receptors and pathways. There are many unresolved questions surrounding CTGF, such as its origins and role in pathological contexts, and this complex landscape will require thorough assessment before we can properly understand the function of CTGF in the myocardium during IR.

**Figure 2:**
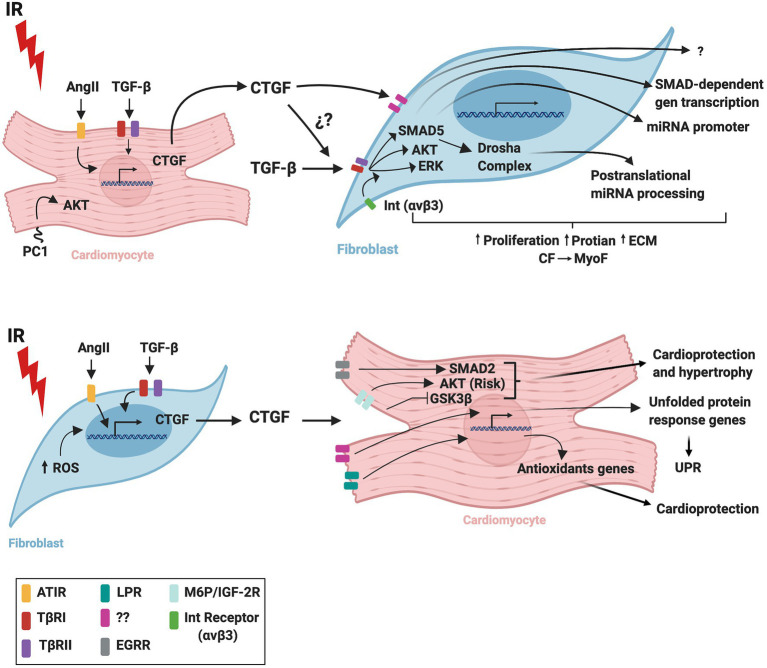
Schematic representation of CTGF crosstalk between cardiomyocytes and fibroblasts during cardiac ischemia/reperfusion. IR, ischemia/reperfusion; AngI, angiotensin II; TGF-β, transforming growth factor beta; CTGF, connective tissue growth factor; ROS, reactive oxygen species; UPR, unfolded protein response; AT1R, angiotensin II receptor type 1; TβRI, transforming growth factor beta receptor I; TβRII, transforming growth factor beta receptor II; LPR, lipoprotein receptor-related protein/α2-macroglobulin receptor; EGFR, epidermal growth factor receptor; M6P/IGF-2R, 6-phosphate/insulin-like growth factor 2 receptor; Int(αvβ3), integrin receptor; CM, cardiomyocytes; CF, cardiac fibroblasts; MyoF, myofibroblasts. The figure was created using BioRender.com.

Figure 2 summarizes probable signaling pathways involved in CM-FB crosstalk regulated by CTGF during IR.

### Cardiac Renin Angiotensin II System

The classic renin angiotensin system (RAS) or circulating RAS is one of the most-studied hormonal systems in the cardiovascular field. This system plays an important role in regulating blood pressure as well as fluid and electrolyte homeostasis in the human body (Wu et al., 2018). However, in pathological scenarios, RAS contributes to hypertension, cardiac hypertrophy, cardiac fibrosis, and IR injury (Dézsi, 2016). Activation of the classic RAS begins with the cleavage of hepatic synthesized-angiotensinogen (AGT) into angiotensin I (Ang I) by renin, followed by transformation of Ang I into angiotensin II (Ang II) by the angiotensin-converting enzyme 1 (ACE1) or the enzyme chymase (Zhou et al., 2012; Froogh et al., 2020). Ang II is considered as the main effector of the classic RAS, and its effect in the cardiovascular system is mediated mainly by Ang II-type 1 (ATR1) and to some extent by Ang II-type 2 (ATR2) receptors, both expressed in CM and CF (Aránguiz-Urroz et al., 2009; Sasa and Kathy, 2014; Lyu et al., 2015; Kilic et al., 2019).

In the cardiovascular system, Ang II induces vasoconstriction and increases myocardial contractility through AT1R activation (Sasa and Kathy, 2014; Kilic et al., 2019) while AT2R stimulation counterbalances these effects, inducing vasodilatation, apoptosis of vascular smooth muscle, and inhibition of cardiac growth and remodeling (Heras et al., 2012; Quadri et al., 2017).

Several signaling pathways are associated with ATR1 activation, such as phospholipase C (PLC)/inositol trisphosphate (IP3)/diacylglycerol (DAG)/Ca^2+^, adenylyl cyclase, tyrosine kinases, tyrosine phosphatases, PLC/phospholipase A2 (PLA2), and JAK/signal transducer and activator of transcription (STAT; De Mello and Jan Danser, 2000; Mendoza Torres et al., 2015). AT1R also activates serine/threonine kinases such as MAPK, including ERK1/2, p38MAPK, and JNK that are implicated in cell growth and hypertrophy (Mehta and Griendling, 2007). On the other hand, AT2R generally exerts its actions through nitric oxide production and activation of phosphatases that inhibit MAPK functions (Gwathmey et al., 2012).

The angiotensin-converting enzyme 2 (ACE2) can either transform Ang II into angiotensin 1–7 (Ang 1–7) or Ang I into angiotensin-1-9 (Ang 1–9). Ang 1–7 can also be processed by the action of neutral-endopeptidase (NEP) and prolyl-endopeptidase (PEP). These peptides, along with ACE2, AT2R, the Mas proto-oncogene receptor, and the Mas-related G protein-coupled receptor member D, are part of the non-canonical RAS, which mediates cardioprotective effects contrary to those of the classic RAS (Santos et al., 2004; Marques et al., 2012; Fattah et al., 2016; Mendoza-Torres et al., 2018; Abdel Ghafar, 2020; Paz Ocaranza et al., 2020).

Many studies have described a local RAS in the heart, whose regulation appears to be independent of the circulating RAS (Ferrario et al., 2016; Silva et al., 2017). The major RAS components, such as renin, ACE1, Ang I, and Ang II, are localized in various areas of the heart, including CF and CM (Paul et al., 2006; Xu et al., 2010; De Mello and Frohlich, 2011). Moreover, the ACE2 system is also found in arterial smooth muscle cells, CM, MF, thoracic aorta, carotid arteries, and veins (Agrawal et al., 2016).

In pathological contexts, chronic cardiac RAS activation has been shown to increase local Ang II levels, which may contribute directly to the cardiac damage observed in IR injury *via* autocrine and/or paracrine communication with neighboring cells (Rodríguez-Lara et al., 2018). Indeed, RAS is locally activated during IR and participates in myocardial repair and further remodeling of the heart. In rats, renin expression and release is increased locally after AMI, and AT1R and ACE2 density is elevated while AT2R density is unchanged (Yang et al., 1998; Burrell et al., 2005; De Mello and Frohlich, 2011; Sabatino et al., 2018). Conversely, decreased AT2R expression was also reported in an AMI rat model (Lax et al., 2004). On the other hand, AGT may be removed from circulation or synthesized by the heart, as observed in rat and mouse models (Paul et al., 2006; Agrawal et al., 2016), suggesting that the heart is capable of synthesizing Ang II locally. In turn, Ang II can contribute to increased infarct size and ROS production by NAPDH oxidase *via* AT1R activation (Dianat et al., 2014; Li et al., 2019).

Locally-activated RAS and Ang II production by CM and/or CF may participate in post-MI repair during the early phase of IR injury (Sun, 2010). Indeed, MI increases AT1R density, which contributes to ECM protein synthesis and further cardiac fibrosis (Díaz-Araya et al., 2015). Moreover, Ang II would have a preferential effect on CF as AT1R density is higher in these cells than in CM (Pellman et al., 2016). Ang II could be also associated with reparative fibrosis after IR *via* ERK signaling-pathway activation (Zhang et al., 2012) and induction of TGF-β and CTGF expression through the ERK, JNK, and p38 MAPK signaling pathways in atrial CF (Gu et al., 2012).

On the other hand, it is possible that Ang II regulates CM and CF expression and/or release of various factors, generating crosstalk between these cells, potentially explaining the role of Ang II in cardiac pathophysiology. Accordingly, fibroblast growth factor 23 (FGF23) overexpression during MI and IR promotes fibrosis and cardiac remodeling (Hao et al., 2016; Leifheit-Nestler et al., 2018). Ang II upregulates FGF23 expression in adult CF (Hao et al., 2016) and CM (Leifheit-Nestler et al., 2018). Moreover, FGF23 is also expressed in CM after MI (Andrukhova et al., 2015), and it was reported that RAS-induced FGF23 in CM stimulates pro-fibrotic factors and CM-CF crosstalk (Leifheit-Nestler and Haffner, 2018; Leifheit-Nestler et al., 2018). Taken together, these reports suggest that Ang II can regulate FGF23 under baseline conditions and during MI. However, more research is required to understand the Ang II-FGF23-mediated crosstalk between CM and CF in the heart during IR.

Given that locally-synthetized Ang II would act on both CM and CF, we can hypothesize that local Ang II is an important factor in the crosstalk between these cells, mediating injury or protection during IR. As this topic is relatively unexplored, further study would be quite useful.

**Figure 3:**
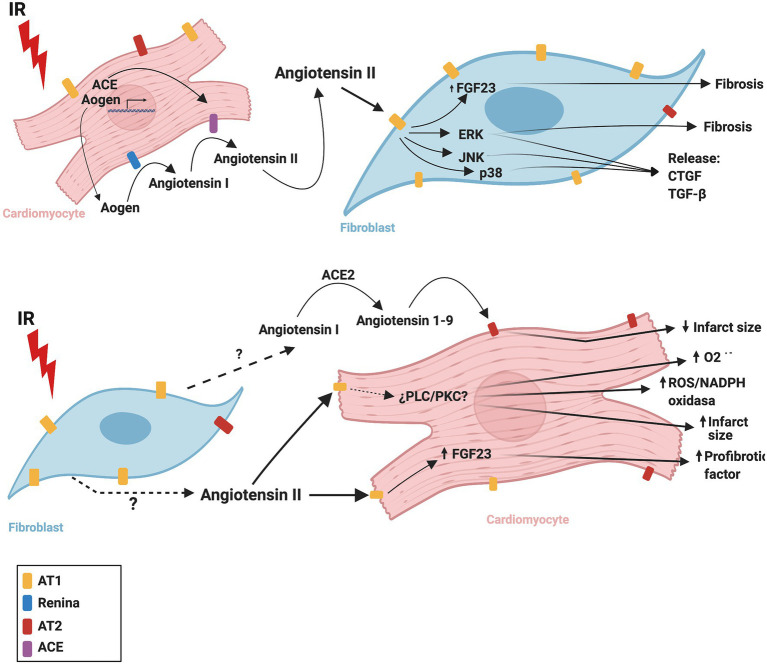
Proposed mechanism of cardiac renin angiotensin system crosstalk between cardiomyocytes and fibroblasts during ischemia/reperfusion. ACE, angiotensin converting enzyme; ACE2, angiotensin converting enzyme 2; AOGEN, angiotensinogen; AT1, angiotensin II receptor type 1; AT2, angiotensin II receptor type 2; CTGF, connective tissue growth factor; ERK, extracellular signal-regulated kinase; FGF23, fibroblast growth factor-23; IR, ischemia reperfusion, JNK, c-Jun N-terminal kinase; NADPH oxidase, nicotinamide adenine dinucleotide phosphate oxidase; O_2_^-.^, superoxide anion; p38, p38 mitogen-activated protein kinase; PLC, phospholipase C; PKC, protein kinase C; TGF-β, transforming growth factor beta. The figure was created using BioRender.com.

Figure 3 summarizes the CM-FB crosstalk regulated by the RAS during cardiac IR.

### Non-coding RNA

Most of the human genome is transcribed into RNA, but only a small fraction of these molecules translate into proteins (Hobuß et al., 2019). In fact, it has been estimated that mRNA accounts for only 2–7% of RNA in mammalian cells (Blahna and Hata, 2012; Palazzo and Lee, 2015; Jaquenod De Giusti et al., 2019). Interestingly, non-coding RNA (ncRNA) can regulate gene expression and function in the cells, where they are produced as well as in recipient cells through horizontal transfer in the extracellular vesicles (EV) exchanged (Jaquenod De Giusti et al., 2019). Thus, it stands to reason that ncRNA have been the focus of recent attempts to clarify mechanisms of cardiovascular physiology and disease (Hobuß et al., 2019).

In terms of cardioprotection, a recent study reported that the long non-coding RNA (lncRNA) AK137033 (named Safe) is expressed in fibroblasts, with increased levels after MI and during TGF-β-induced cardiac fibrosis (Hao et al., 2019). Notably, inhibition of Safe by shRNA injection improved left ventricular functional recovery in mice subjected to MI. In addition, Safe inhibition impaired the CF-to-MF differentiation provoked by TGF-β in adult mice. In terms of the underlying mechanisms, the authors concluded that Sfrp2 mRNA – a gene essential in regulating cardiac fibrosis – attaches to Safe mRNA through complementary binding at the 3′-end to provide mutual stabilization (Hao et al., 2019). In addition, the authors found that Safe was also expressed in adult CM, albeit at levels much lower than in CF, suggesting a potential crosstalk mechanism for regulation of CF-induced fibrosis; future studies should explore this topic. Safe is, therefore, an attractive therapeutic target for limiting MI-elicited fibrosis and thereby attenuating heart failure.

Another study shed light on the potential mechanisms by which lncRNA may be communicated from one cell to another, showing that CM subjected to H/R release EV that can induce the expression of pro-fibrotic genes in CF (Kenneweg et al., 2019). Interestingly, the authors found that the lncRNA ENSMUST00000122745 and Neat1 are both sensitive to hypoxia and are enriched in small and large EV, respectively. Moreover, Neat1 was found to be a key for CF survival and function, as well as cardiac activity after MI (Kenneweg et al., 2019). Another study indicated that hypoxic adult rat CM release small EV that impair CF function while increasing apoptosis (Wang and Zhang, 2020). This work reported that the lncRNA AK139128 was enriched in EV isolated from hypoxic CM, and co-culture experiments indicated that EV derived from hypoxic CM may augment AK139128 expression in CF. Furthermore, the authors also showed that AK139128 carried by hypoxic EV impaired CF migration, inhibited proliferation, and increased apoptosis (Wang and Zhang, 2020). Thus, these studies suggest that EV are important players to consider in lncRNA-mediated crosstalk between CM and CF. The lncRNA AK139128 may be involved in this communication during IR, potentially as a regulator of cardiac remodeling.

Other lncRNA have also been reported to participate in cardiac IR. MI-associated transcript (MIAT) has been linked to increased injury (Ishii et al., 2006; Zangrando et al., 2014; Liu et al., 2019a), fibrosis, and adverse remodeling (Qu et al., 2017) after AMI; cardiac apoptosis-related lncRNA (Carl), mitochondrial dynamic related lncRNA (Mdrl), and five prime to Xist (Ftx) downregulation are also associated with apoptosis during IR (Wang et al., 2014a,b; Long et al., 2018). Furthermore, cardiac autophagy inhibitory factor (CAIF) downregulation was associated with increased cardiac injury (Liu et al., 2018a); necrosis-related factor (NRF) upregulation during IR with increased necrosis (Wang et al., 2016a); and Wisp2 super-enhancer-associated RNA (Wisper) upregulation with fibrosis and cardiac remodeling (Micheletti et al., 2017). Most of these lncRNA target miRNA to regulate cell response. However, their potential contributions to paracrine signaling and CM-CF crosstalk have not been thoroughly studied.

Among miRNA, miR-221 has been recently found to exert an important cardioprotective effect (Zhou et al., 2019). This miRNA shields H9c2 cells from the deleterious consequences of H/R both by reducing apoptosis and autophagy but protects CF only by inhibiting autophagy. In addition, miR-221 inhibits CF-to-MF differentiation, suggesting that it may ameliorate reactive cardiac fibrosis. The cardioprotective effects of miR-221 were confirmed by *in vivo* IR experiments in rats, whereby the administration of this miRNA reduced infarct size and fibrosis, improving left ventricular function (Zhou et al., 2019). miR-223 is another relevant miRNA in the province of cardioprotection. miR-223 levels are higher in CF than CM, and expression is increased in response to TGF-β1 (Liu et al., 2018b). In this context, miR-223 promotes CF migration and proliferation as well as collagen I, collagen III, and α-SMA expression. These effects appear to be mediated *via* RAS p21 protein activator 1 (RASA1). Additionally, miR-223 inhibition reduced cardiac fibrosis and preserved left ventricular function after MI in rats *in vivo* (Liu et al., 2018b). Similar to what has been observed with lncRNA, EV have also been found to transport miRNA. Post-conditioned CF release EV that can carry miR-423-3p, improving the viability of H9c2 cells subjected to H/R. Moreover, EV from post-conditioned CF reduced infarct size after *in vivo* MI in rats, and miR-423-3p inhibition prevented the protective effect of post-conditioning (Luo et al., 2019), possibly indicting that this miRNA makes a crucial contribution to cardioprotection by horizontal transfer between CF and CM. While *in vitro* studies suggest that the protective effects of miR-423-3p are mediated by its targeting of Ras-related protein Rap-2c (RAP2C), these findings should be replicated in primary CM (Luo et al., 2019).

In addition to miR-423-3p, many other miRNA have been reported to play roles in IR through cardiac cell crosstalk. miR-208a (Yang et al., 2018) and miR-195 (Morelli et al., 2020) are upregulated and released from CM in EV during IR, inducing fibroblast proliferation and differentiation into MF as well as fibrosis after MI. On the other hand, miR-21 from CM EV, one of the most-studied miRNA, had been demonstrated a protective effect against MI and fibrosis (Zhu and Fan, 2012; Chen et al., 2019), reflecting its function in cardiac crosstalk during IR. An extensive review of miRNA and their role in cardiac disease and MI is available in the scientific literature (Ottaviani et al., 2018), although more studies will be needed to completely explain their contributions to cardiac cell-to-cell communication.

Other ncRNA may also be important in cardioprotection. A novel study found that the circular RNA CircNFIB may reduce cardiac fibrosis by sponging miR-433 (Zhu et al., 2019), suggesting a new therapeutic target for limiting AMI-induced cardiac fibrosis. Another insight is related to novel miRNA regulation mechanisms; it was recently reported that miR-1 can be oxidized by ROS and consequently trigger cardiac hypertrophy (Seok et al., 2020). However, this type of regulation has not been described during IR. This finding may be a game-changer in our understanding of the role of ncRNA in cardiovascular disease. Therefore, the potential of oxidative modifications to alter the function of ncRNA and its potential participation in cardioprotection merits further research.

**Figure 4:**
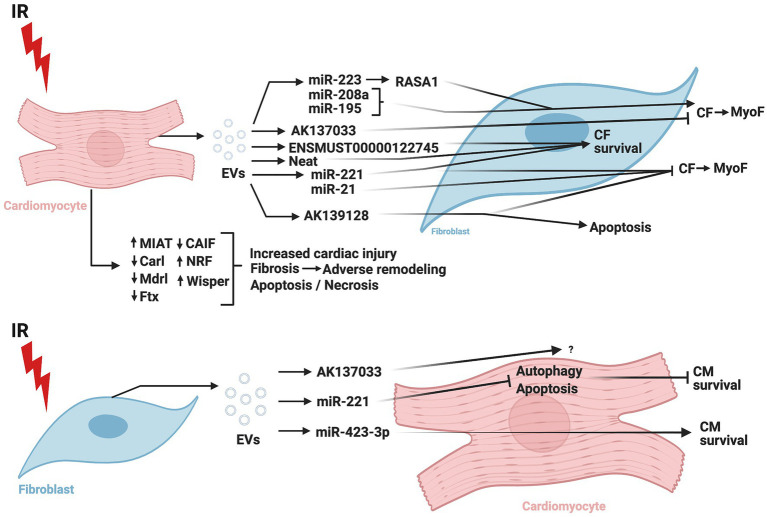
Role of non-coding RNAs in cardiomyocyte-cardiac fibroblast crosstalk in ischemia/reperfusion injury. IR, ischemia/reperfusion; EV, extracellular vesicles; CM, cardiomyocytes; CF, cardiac fibroblasts; MyoF, myofibroblasts; MIAT, myocardial infarction-associated transcript; Carl, cardiac apoptosis-related lncRNA; Mdrl, mitochondrial dynamic related lncRNA; Ftx, five prime to Xist; CAIF, cardiac autophagy inhibitory factor; NRF, necrosis-related factor; Wisper, Wisp2 super-enhancer-associated RNA; miR, micro-RNA; RASA1, RAS p21 protein activator 1. The figure was created using BioRender.com.

Figure 4 summarizes the CM-CF crosstalk regulated by ncRNA during cardiac IR.

## Pharmacological Perspectives in the Context of Crosstalk

Despite extensive efforts to generate therapeutics and drugs to mitigate the myocardial injury and pathological remodeling caused by IR and cardiac infarct, to date no approach has been satisfactorily effective. The major therapies available are focused on avoiding the oxidative damage and excessive inflammatory response induced by IR. Recent studies have evaluated drugs with well-established safety and efficacy as potential therapies in the post-infarcted heart. These drugs may be older, but there is still there is a marked lack of knowledge regarding their pharmacological mechanisms of action.

Communication between CM and CF represents an interesting therapeutic target since the cellular responses associated with various molecules that mediate cell communication are closely related to the healing process and eventual fibrosis after MI.

Simvastatin (a cholesterol synthesis inhibitor) and valsartan (an angiotensin type 1 receptor blocker) have been demonstrated to prevent MI-induced heart dysfunction and fibrosis. Notably, these effects were associated with decreased TGF-β1 concentration and SMAD3 activation (Sui et al., 2015; Xiao et al., 2016), suggesting a probable multifactorial mechanism of action. Indeed, valsartan may simultaneously inhibit Ang II and TGF-β1 during IR. Similar data were obtained with post-AMI treatment with pyridostigmine, vitamin D, and linagliptin in pre-clinical murine models (Lu et al., 2014; Le et al., 2018; Yamaguchi et al., 2019). TGF-β1 inhibition, therefore, may underlie this cardioprotective function.

GW788388, a specific TβRI inhibitor, prevented IR-dependent cardiac damage in a rat MI model (Tan et al., 2010), decreasing CMF accumulation, cardiac fibrosis, CM hypertrophy, and systolic dysfunction (Tan et al., 2010). Interestingly, early inhibition of TGF-β1 (first 24h) after AMI with a blocking antibody worsens the pathological consequences of IR injury, while later inhibition ameliorated these effects (Ikeuchi et al., 2004). TGF-β1 may have a dual role in IR-injury, initially as an anti-inflammatory and cardioprotective agent, and later as a pro-fibrotic molecule that impairs cardiac function and survival. Therefore, there may be a treatment window after MI during which TGF-β1 may offer a therapeutic target for cardioprotection.

On the other hand, tranilast, an anti-allergic and TGF-β expression inhibitor, decreased the profibrotic effects induced by IR injury in a preclinical study (See et al., 2013). In a Phase III clinical study, the drug decreased the number of AMI events (Holmes et al., 2002). On the other hand, pirfenidone decreases TGF-β1 signaling. This drug is approved to treat idiopathic pulmonary fibrosis and is being tested in a Phase II clinical trial in patients with left ventricular dysfunction (Nguyen et al., 2010). In any case, it has yet to be tested during MI.

Use of monoclonal antibodies is another interesting strategy under exploration in various pathologies. While CTGF monoclonal antibody treatment apparently improved survival and reduced left ventricular dysfunction, hypertrophy, and fibrosis after cardiac ischemia and during post-AMI remodeling, CTGF antagonism had no effect on CM viability and infarct size following IR injury (Vainio et al., 2019). Indeed, while the CTGF antibody FG-3019 (pamrevlumab) is in Phase 3 clinical development for the treatment of idiopathic pulmonary fibrosis (Sgalla et al., 2020) and locally-advanced unresectable pancreatic cancer (ClinicalTrials.gov Identifier: NCT03955146) and in Phase 2 clinical development for Duchenne muscular dystrophy (ClinicalTrials.gov Identifier: NCT02606136) and COVID-19 (ClinicalTrials.gov Identifier: NCT04432298), it has never been assayed as a cardioprotective drug after MI injury. Further studies are needed to establish whether CTGF antagonism provides a novel therapy for AMI patients that might prevent the cardiac remodeling that leads to HF.

As mentioned, valsartan may mitigate cardiac injury and remodeling after MI through AT1R blocking. Moreover, several ACE inhibitors have been broadly studied and used to prevent the consequences of IR injury *in vivo* (Strauss and Hall, 2016; Bahit et al., 2018; Messerli et al., 2018). However, the most promising therapies related to local and systemic RAS axis inhibition involve in the regulation of the counter-regulatory system (non-canonical RAS components).

Ang (1–9) infusion decreased infarct size during reperfusion (Mendoza-Torres et al., 2018), and *in vivo* Ang (1–9) gene transfer exerted cardioprotective effects in a murine model of AMI (Fattah et al., 2016). These promising results will stimulate more pre-clinical studies to evaluate this peptide as post-infarction therapy. Additionally, Ang (1–7) administration has been demonstrated to improve cardiac function and reperfusion-induced arrhythmias after MI (Santos et al., 2004; Marques et al., 2012). This evidence supports the idea of potentiating the effects of the non-canonical RAS to protect the myocardium from IR injury.

On the other hand, the AT2R agonist compound 21 (C21) improved cardiac function and prevented cardiac remodeling in rats subjected to permanent ligation of the left coronary artery by decreasing matrix metalloproteinase 2 and 9 and TGF-β1 expression in the left ventricle (Lauer et al., 2014). The actions of both CM and CF are strictly coordinated, and evidence shows that canonical and non-canonical RAS components are expressed in both cell types. The shorter peptides, derived from proteolytic processing of angiotensin I and II peptides, have biological activity and effects opposite those mediated by Ang II, which might represent a novel strategy for reducing IR injury in the heart.

Finally, one of the most exciting and attractive pharmacological research avenues in MI therapy involves ncRNA. This approach offers massive potential for thorough analysis of the pathophysiological cross-talk between CF and CM in the setting of IR injury and, therefore, the possibility of new targets or agents to attenuate MI in humans. Nonetheless, while ncRNA are therapeutically promising, pharmacological targeting of ncRNA may be difficult. For example, most miRNA deemed potential therapeutic agents remain in the early phases of clinical trials, and whether miRNA demonstrate suitable efficacy and safety for widespread use in humans remains to be seen. Extensive preclinical data regarding the safety and basic characteristics of such a drug, as well as a Phase I clinical trial protocol, are required to apply for authorization for a clinical trial. Therefore, there is a long road ahead before agents that act as miRNA mimics or inhibitors might become available (Chakraborty et al., 2021). Anti-miRNA oligonucleotides are currently administered using intravenous and subcutaneous routes, given their low absorption after oral administration, but further research is still needed to accurately assess the pharmacokinetics of miRNA and develop formulations with adequate half-lives (Chakraborty et al., 2021).

Pharmacological agents targeting lncRNA offer a promising therapeutic strategy, but the safety of this therapy must still be assessed, given that most of the evidence is preclinical. Off-target effects may cause adverse reactions. Thus, optimal delivery and specific targeting is crucial to ensure that only the specific lncRNA is targeted (Chen et al., 2021). Currently, drugs that can target miRNA exert their effects through well-known mechanisms, and this approach remains the focus of most clinically-relevant studies involving ncRNA. Eventually, a more comprehensive exploration of lncRNA will likely take on a more central role (Matsui and Corey, 2017). Nevertheless, despite the exciting possibilities surrounding these therapies, much RNA research has been found to be irreproducible. Therefore, drug development involving ncRNA must proceed with caution. An in-depth evaluation of the relationship between molecular mechanisms and biological effects (Matsui and Corey, 2017) will be necessary before ncRNA-based clinical pharmacology in the context of CF-CM cross-talk can become a reality for MI treatment.

## New Perspectives

Cellular crosstalk is a crucial mechanism for regulating cardiac physiology. The importance of this mechanism in many pathologies is evident. However, our understanding of the causal mechanisms remains at a preliminary stage due, at least in part, to redundant signaling pathways and the challenge of developing precise and reliable experimental models to assess interactions between different cell types including 2D and 3D models. More extensive knowledge of crosstalk between different types of cardiac cells is crucial for explaining IR-/AMI-induced myocardial damage and remodeling, as well as the progression towards cardiac hypertrophy and heart failure, which is a highly-relevant public health problem.

Recently, new approaches to understanding CM-CF communication during IR and remodeling have emerged. For one, proteasome inhibition has been effective against IR injury in preclinical research (Sanchez et al., 2016; Olmedo et al., 2020). Indeed, a recent study that showed that the proteasome inhibitor ixazomib reduced infarct size and improved recovery of left ventricular function after *ex vivo* MI in rats (Sánchez et al., 2020). A novel study has shed more light on the effects of proteasome inhibition, which may involve a complex interplay between cardiac cells. In that study, proteasome inhibition provoked apoptosis in neonatal mouse CM. However, when the proteasome was inhibited in a heterogenous culture of CM, CF, cardiac endothelial cells, and cardiac vascular smooth muscle cells, apoptosis was significantly reduced in the CM while remaining at typical levels in the other cardiac cells. This finding suggests a crucial protective role for non-CM cells, such as CF, in order to promote CM survival after proteasome inhibition. Moreover, this study found that brain natriuretic peptide is released from CM upon proteasome inhibition, signaling other cardiac cells to prevent proteasome inhibition-induced CM death *via* a paracrine function (Guo et al., 2020). The mechanisms responsible for this crosstalk between CM and other cardiac cells, such as CF, remain to be elucidated. While the aforementioned study was not performed under IR conditions, it hints at the provocative idea that proteasome inhibition protects the myocardium from MI by a mechanism that involves sacrificing fibroblasts and other cardiac cells in order to save CM. This idea certainly merits further research.

While several pre-clinical drugs have been shown to protect against IR injury, these effects were, unfortunately, not replicated in clinical trials.

Future studies must clarify the mechanisms that underlie crosstalk between cardiac cells, as well as the signaling pathways involved in these interactions, to support clinical strategies and drugs that effectively avoid or reduce AMI-induced myocardial damage and ventricular dysfunction.

We hope that this review contributes to understanding the importance of CM-CF communication during IR and remodeling, emphasizing the most-studied molecules and signaling pathways to date, as well as potential pharmacological approaches based on these findings. However, several other molecules and cell types are involved in cardiac cell communication and play important pathophysiological roles during MI injury including inflammatory factors, molecules transported in extracellular vesicles (such as exosomes), and other cardiac cells. We acknowledge as a study limitation that we were unable to address these factors here. Further research will be necessary for a comprehensive understanding of communication between CM and CF in cardiac tissue.

## Author Contributions

RF-V, IO, and ZP edited the manuscript. RF-V created the figures. ZP designed and coordinated the work. All authors contributed to the article and approved the submitted version.

## Conflict of Interest

The authors declare that the research was conducted in the absence of any commercial or financial relationships that could be construed as a potential conflict of interest.

## Publisher’s Note

All claims expressed in this article are solely those of the authors and do not necessarily represent those of their affiliated organizations, or those of the publisher, the editors and the reviewers. Any product that may be evaluated in this article, or claim that may be made by its manufacturer, is not guaranteed or endorsed by the publisher.
